# Association between the neutrophil-to-lymphocyte ratio and in-hospital mortality in patients with chronic kidney disease and coronary artery disease in the intensive care unit

**DOI:** 10.1186/s40001-024-01850-3

**Published:** 2024-04-30

**Authors:** Jingjing Luo, Yufan Zhou, Yu Song, Dashuai Wang, Meihong Li, Xinling Du, Jihong Kang, Ping Ye, Jiahong Xia

**Affiliations:** 1 Department of Cardiovascular Surgery, Union Hospital, Tongji Medical College, Huazhong University of Science and Technology, Wuhan, 430022 China; 2 Department of Cardiovascular Surgery, The First Affiliated Hospital of Zhengzhou University, Henan Province, 450052 China; 3 Department of Physiology and Pathophysiology, School of Basic Medical Sciences, Peking University Health Science Center, Beijing, 100038 China; 4 Department of Cardiology, The Central Hospital of Wuhan, Tongji Medical College, Huazhong University of Science and Technology, Wuhan, 430014 China

**Keywords:** Neutrophil-to-lymphocyte ratio, Coronary artery disease, Chronic kidney disease, In-hospital mortality, MIMIC-IV database

## Abstract

**Background:**

The objective of this study was to investigate the correlation between neutrophil-to-lymphocyte ratios (NLR) and the risk of in-hospital death in patients admitted to the intensive care unit (ICU) with both chronic kidney disease (CKD) and coronary artery disease (CAD).

**Methods:**

Data from the MIMIC-IV database, which includes a vast collection of more than 50,000 ICU admissions occurring between 2008 and 2019, was utilized in the study and eICU-CRD was conducted for external verification. The Boruta algorithm was employed for feature selection. Univariable and multivariable logistic regression analyses and multivariate restricted cubic spline regression were employed to scrutinize the association between NLR and in-hospital mortality. The receiver operating characteristic (ROC) curves were conducted to estimate the predictive ability of NLR.

**Results:**

After carefully applying criteria to include and exclude participants, a total of 2254 patients with CKD and CAD were included in the research. The findings showed a median NLR of 7.3 (4.4, 12.1). The outcomes of multivariable logistic regression demonstrated that NLR significantly elevated the risk of in-hospital mortality (OR 2.122, 95% confidence interval [CI] 1.542–2.921, *P* < 0.001) after accounting for all relevant factors. Further insights from subgroup analyses unveiled that age and Sequential Organ Failure Assessment (SOFA) scores displayed an interactive effect in the correlation between NLR and in-hospital deaths. The NLR combined with traditional cardiovascular risk factors showed relatively great predictive value for in-hospital mortality (AUC 0.750).

**Conclusion:**

The findings of this research indicate that the NLR can be used as an indicator for predicting the likelihood of death during a patient’s stay in the intensive care unit, particularly for individuals with both CAD and CKD. The results indicate that NLR may serve as a valuable tool for assessing and managing risks in this group at high risk. Further investigation is required to authenticate these findings and investigate the mechanisms that underlie the correlation between NLR and mortality in individuals with CAD and CKD.

**Supplementary Information:**

The online version contains supplementary material available at 10.1186/s40001-024-01850-3.

## Introduction

Chronic kidney disease (CKD) has a substantial impact on healthcare systems globally, leading to increased illness and death rates. The occurrence of coronary artery disease (CAD) is much higher in individuals with CKD compared to the overall population, often leading to a negative prognosis [1]. Furthermore, the causes of vascular ailments in individuals with concomitant CAD and CKD are more complicated and involve variables other than atherosclerosis and inflammation [2]. Therefore, it is critical to ascertain whether inflammation and unfavorable outcomes are still associated in this group.

Neutrophil-to-lymphocyte ratios (NLR) have the potential to be innovative biomarkers of the underlying inflammatory process [3]. They may also be excellent predictors in patients with a variety of diseases including immune-related adverse outcomes in cancer patients [4], ischemic stroke [5], cardiovascular disease [6], the progression of end-stage renal disease (ESRD) [7] and hematological disorders [8]. Lately, NLR has also been considered an option for astronaut immunological state monitoring [9]. The inflammatory response is a key mechanism of coronary atherosclerosis in patients with normal renal function. Several prior clinical studies have shown that NLR is linked to cardiovascular disease severity, in-hospital mortality, cardiovascular morbidity, and long-term prognosis in patients with cardiovascular disease [10–13].

However, the role of inflammation in disease incidence and progression varies from normal kidney function people and CKD patients, especially combined with CAD. Moreover, there was still uncertainty regarding the relationship between these ICU patients’ NLR and unfavorable hospital outcomes. Using information from a publicly accessible database, this research assessed the significance of the NLR in predicting the probability of in-hospital mortality in the intensive care unit for individuals with CKD and CAD.

## Methods

### Data sources and extraction

The research employed the Medical Information Mart for Intensive Care-IV (MIMIC-IV), an openly accessible repository containing data from more than 50,000 admissions to the intensive care unit (ICU) at Beth Israel Deaconess Medical Center in Boston, Massachusetts, spanning from 2008 to 2019 [14]. The MIMIC-IV dataset covers a vast array of data, comprising demographic details, vital indicators, examination outcomes, and diagnoses, recorded using the ICD-9 and ICD-10 codes of the International Classification of Diseases. Furthermore, the research conducted the Telehealth Intensive Care Unit Collaborative Research Database (eICU-CRD) for external verification, which is a publicly available multicenter database over 200,000 ICU admissions from 208 hospitals. Jingjing Luo, the author, successfully acquired the necessary certification (certification number 12890900) to access the databases and retrieve the essential variables for the study. Since the databases guaranteed the confidentiality of patients, obtaining individual patient consent was considered unnecessary. The research included individuals who were diagnosed with both CAD and CKD using ICD-9 and ICD-10 codes from the website https://www.icd.who.int/browse10/2019/en. CAD was defined according to the codes 410–411 (ICD-9) and I20–I21 (ICD-10), and CKD was defined based on the codes 585, V451 (ICD-9) and N18, Z99.2 (ICD-10) [15]. Individuals who had less than 6 h of ICU stay were under the age of 18, lacked baseline creatinine readings, or had over 30% missing data, or the absence of lymphocyte and neutrophil values, were excluded. In cases of multiple admissions, only data from the initial admission were considered. Using pgAdmin PostgreSQL tools (version 1.22.1), we extracted data from the two databases including demographic data, lab findings, vital signs recorded hourly, existing health conditions, prescribed drugs, surgical operations, details of ICU stay, and records of mortality during hospitalization. The dataset utilized the 'mice' package in R for imputing missing data [16].

### Feature selection

After establishing the significance of the study variables, an important step was to select the features to simplify the dataset. The Boruta algorithm, which played a key role in this process (Additional file 1: Materials S1), employed the random forest classifier method, thereby significantly impacting the procedure. The algorithm created shadow features, duplicating the original dataset, and compared the *Z*-scores between genuine and shadow features in each iteration. Features with a *Z*-score higher than the maximum *Z*-score for shadow features were kept, while those below were removed [17].

Afterward, the R package called ‘ingredients’ was employed to calculate various metrics including root mean square error (RMSE) loss following permutations, the percentage rise in mean square error (%IncMSE), and the growth in node purity (IncNodePurity). These metrics were used to evaluate the significance of variables in the random forest model [18]. RMSE and MSE serve as common indicators for evaluating machine learning model performance, with lower values indicating better fit. %IncMSE reflects the percentage increase in MSE when a feature is added, ranging from 0 to positive infinity. A small % of IncMSE implies minimal model performance change, while a larger value suggests a substantial change after feature addition [19]. Node purity measures the classification purity of nodes in the decision tree, indicating the extent to which a feature enhances node purity when added. A higher IncNode Purity value denotes a greater contribution to node classification, resulting in increased node purity [20].

Combining these results with clinical insights and addressing variable collinearity, the most pertinent variables associated with in-hospital mortality were incorporated into the final model.

### Statistical analysis

The hospital stay determined the stratification of patients into two groups according to their survival status. Comparisons between categorical variables, expressed as percentages, were conducted using either Fisher’s exact probability method or Chi-square tests. The Wilcoxon rank sum test was used to evaluate the medians of continuous variables, which were represented as interquartile ranges.

The NLR value was determined by dividing the neutrophils (NEU, 10^9^/L) by the lymphocytes (LYM, 10^9^/L), which were the highest absolute values recorded for NEU and LYM throughout the patient's stay in the hospital. Univariable and multivariable logistic regression analyses were conducted to investigate the correlation between NLR and the risk of in-hospital mortality. Model 1 exclusively featured NLR without additional adjustments. Gender and age modifications were integrated into Model 2. Taking into account feature selection results and adjustments made according to clinical expertise, Model 3 was fully fine-tuned. Moreover, a trichotomous multivariable restricted cubic spline (RCS) regression was utilized to investigate possible non-linear associations between NLR and mortality during hospitalization. Age, gender, SOFA score, diabetes, dialysis, and acute coronary syndrome (ACS) were taken into account in subgroup analyses, and interactions were assessed using P values. In addition, receiver operating characteristic (ROC) curves were conducted to compare NLR with traditional cardiovascular scores (age, sex, max systolic blood pressure, treatment for blood pressure, diabetes, body mass index (BMI), dialysis) and area under curves (AUC) were calculated. The Delong’s test was performed to compare different ROC curves.

SPSS (version 26.0, IBM) and R (version 4.1.3, Austria) were utilized for all statistical analyses. A significance level of less than 0.05 was established for statistical significance, using a two-sided P value.

## Results

### Baseline characteristics

**Fig. 1:**
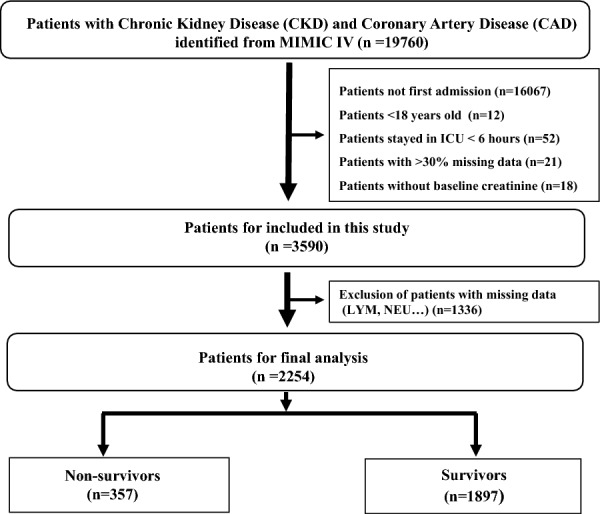
The selection flowchart of CKD and CAD patients from the MIMIC-IV database. *MIMIC* Medical Information Mart for Intensive Care

The study included 2254 patients with chronic kidney disease (CKD) and coronary artery disease (CAD) from MIMIC-IV, based on the criteria for inclusion and exclusion (Fig. 1). The median NLR was 7.3 (4.4, 12.1). Out of the total number of patients, 357 individuals (15.8%) passed away during their hospital stay, whereas the remaining 1897 patients managed to survive. The baseline characteristics are summarized in Table 1 and Additional file 2: Table S1. The NLR values and baseline serum creatinine were higher in patients who passed away while receiving hospital care, and they also had increased risks for myocardial infarction (*P* < 0.001).

**Table 1:** Baseline characteristic for patients included in the study divided by in-hospital situation

	Overall	Survivor	Non-survivor	*P*-Value
*N*	2254	1897	357	
Age	76.0 [68.0,83.0]	75.0 [67.0,82.0]	79.0 [70.0,85.0]	< 0.001
Female, *n* (%)	725 (32.2)	604 (31.8)	121 (33.9)	0.484
los_icu	2.6 [1.3,4.8]	2.5 [1.3,4.3]	3.3 [1.3,6.7]	0.004
scr_baseline	1.4 [1.1,2.0]	1.3 [1.0,1.9]	1.6 [1.1,2.5]	< 0.001
myocardial_infarct, *n* (%)	1506 (66.8)	1229 (64.8)	277 (77.6)	< 0.001
ACS, *n* (%)	644 (28.6)	534 (28.1)	110 (30.8)	0.338
Aspirin, *n* (%)	1926 (85.4)	1651 (87.0)	275 (77.0)	< 0.001
Clopidogrel, *n* (%)	641 (28.4)	550 (29.0)	91 (25.5)	0.2
Statin, *n* (%)	1888 (83.8)	1646 (86.8)	242 (67.8)	< 0.001
beta_blocker, *n* (%)	1690 (75.0)	1484 (78.2)	206 (57.7)	< 0.001
Warfarin, *n* (%)	610 (27.1)	557 (29.4)	53 (14.8)	< 0.001
troponin_max	0.3 [0.1,1.1]	0.2 [0.1,0.9]	0.6 [0.2,2.1]	< 0.001
wbc_max	15.6 [11.6,20.9]	15.2 [11.4,20.0]	19.8 [14.3,26.5]	< 0.001
rbc_max	3.7 [3.3,4.2]	3.7 [3.4,4.2]	3.7 [3.2,4.2]	0.016
platelet_max	260.0 [194.0,345.0]	263.0 [199.0,350.0]	236.0 [158.0,317.0]	< 0.001
alt_max	29.0 [17.0,69.0]	26.0 [16.0,52.0]	76.0 [29.0,316.0]	< 0.001
ast_max	44.5 [25.0,109.0]	39.0 [24.0,85.0]	136.0 [47.0,668.0]	< 0.001
bun_max	56.0 [38.0,82.0]	52.0 [36.0,77.0]	76.0 [52.0,106.0]	< 0.001
inr_max	1.5 [1.3,2.4]	1.5 [1.3,2.2]	2.0 [1.5,3.4]	< 0.001
pt_max	16.8 [14.2,25.5]	16.2 [13.9,23.9]	22.1 [16.3,36.1]	< 0.001
ptt_max	53.0 [33.1,114.2]	47.6 [32.4,104.5]	79.7 [41.3,148.5]	< 0.001
glucose_mean	132.8 [113.2,164.0]	129.9 [112.2,159.4]	153.8 [124.0,195.8]	< 0.001
SOFA	6.0 [4.0,9.0]	6.0 [4.0,8.0]	10.0 [7.0,13.0]	< 0.001
BMI	28.1 [24.4,32.8]	28.1 [24.5,32.8]	28.0 [24.0,33.3]	0.703
sbp_min	84.0 [73.0,93.0]	85.0 [76.0,95.0]	70.0 [54.0,83.0]	< 0.001
dbp_mean	58.2 [52.5,64.3]	58.6 [53.0,64.7]	56.3 [50.2,62.2]	< 0.001
hr_mean	80.2 [71.9,89.2]	79.3 [71.6,87.9]	87.3 [75.5,97.0]	< 0.001
spo2_min	90.0 [85.0,92.0]	90.0 [87.0,93.0]	83.0 [74.0,90.0]	< 0.001
NLR	7.3 [4.4,12.1]	6.7 [4.1,10.9]	11.5 [7.4,19.1]	< 0.001
LY_abs_max	1.4 [0.9,2.0]	1.4 [0.9,2.0]	1.1 [0.8,1.8]	< 0.001
NEU_abs_max	10.0 [6.8,14.3]	9.5 [6.5,13.5]	13.8 [9.7,20.0]	< 0.001
LYM%_max	12.9 [8.2,19.8]	13.8 [9.0,20.3]	8.9 [5.9,13.4]	< 0.001
NEU%_max	82.0 [74.6,88.0]	81.0 [73.8,87.1]	86.3 [80.4,90.8]	< 0.001

*NLR* neutrophil-to-lymphocyte ratio, *scr* serum creatinine, *ACS* acute coronary syndrome, *max* maximum, *min* minimum, *alt* alanine aminotransferase, *ast* aspartate aminotransferase, *inr* International Normalized Ratio, *pt* prothrombin time, *ptt* partial thromboplastin time, *SOFA* sequential organ failure assessment, *hr* heart rate, *spo2* oxyhemoglobin saturation, *bun* blood urea nitrogen, *NEU* neutrophils, *LYM* lymphocytes, *abs* absolute

### Feature selection

**Fig. 2:**
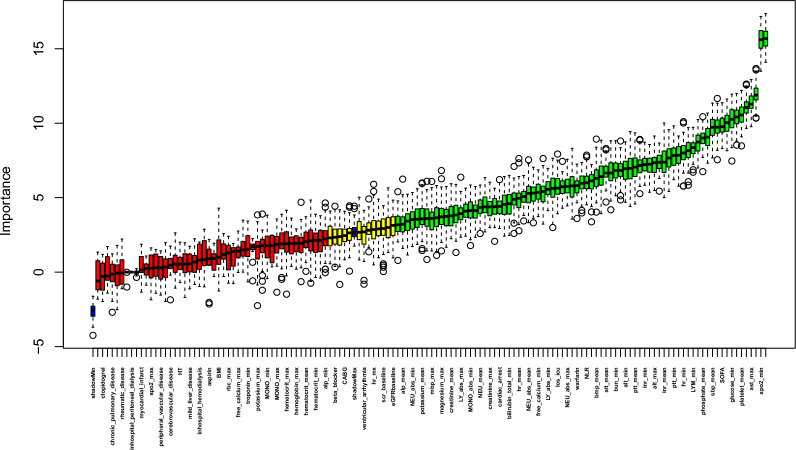
The Boruta algorithm conducted the feature selection for the relationship between NLR and in-hospital mortality. The horizontal axis shows the name of each variable, while the vertical axis represents the Z-value of each variable. The box plot depicts the Z-value of each variable in the model calculation, with green boxes representing important variables, yellow representing tentative attributes, and red representing unimportant variables. *NLR* neutrophil-to-lymphocyte ratio, *scr* serum creatinine, *eGFR* estimated glomerular filtration rate, *ACS* acute coronary syndrome, *HT* hypertension, *max* maximum, *min* minimum, *WBC* white blood cell, *RBC* red blood cell, *ALT* alanine aminotransferase, *INR* International Normalized Ratio, *PT* prothrombin time, *SOFA* sequential organ failure assessment, *HR* heart rate, *SpO*_*2*_ oxyhemoglobin saturation

**Fig. 3:**
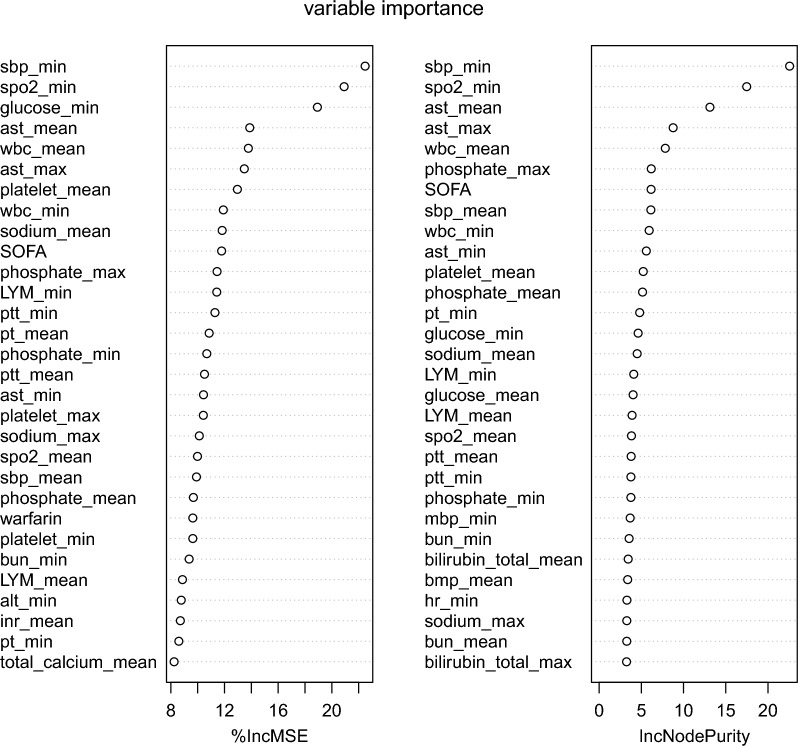
Top 30 important variables for the relationship between NLR and in-hospital mortality based on the percentage of the increase in mean square error and the increase in node purity. *NLR* neutrophil-to-lymphocyte ratio, *max* maximum, *min* minimum, *WBC* white blood cell, *ALT* alanine aminotransferase, *PT* prothrombin time, *SOFA* sequential organ failure assessment, *SpO*_*2*_ oxyhemoglobin saturation, *%IncMSE* the percentage of the increase in mean square error, *IncNodePurity* the increase in node purity

The Boruta algorithm confirmed 77 of 137 variables that were the most related to in-hospital death (Fig. 2, and Additional file 2: Table S2). Additional file 2: Table S3 displays the selection of the top 30 variables deemed most significant based on RMSE loss following permutations, %IncMSE, and IncNodePurity. The final complete adjustment model selected factors based on their Z-scores in the Boruta analysis surpassing the shadow features. Additionally, factors were included if they had the greatest matched effect and importance in the model, as indicated by RMSE loss after permutations, %IncMSE, and IncNodePurity. These criteria are illustrated in Fig. 3 and Additional file 3: Figure S1. Furthermore, factors were considered if they aligned with previous findings and clinical constraints, such as ACS. After eliminating collinearity, the fully adjusted model included a total of 15 variables: NLR, age, gender, sbp_min, spo2_min, ast_max, phosphate_max, wbc_mean, SOFA, glucose_min, bun_min, platelet_mean, pt_min, ptt_mean, and ACS.

### NLR and in-hospital mortality

**Fig. 4:**
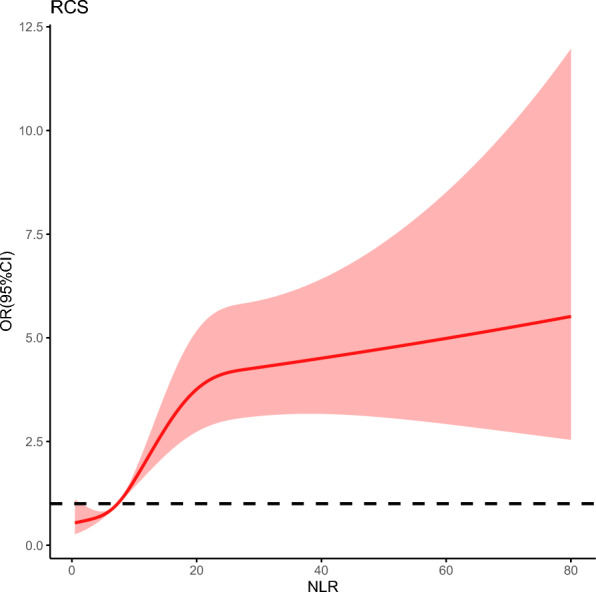
The nonlinear association between the NLR and in-hospital mortality after full adjustment via multivariable RCS regression. The cutoff value in the plot of in-hospital death risk was 7.32. NLR, neutrophil-to-lymphocyte ratio; RCS, restricted cubic spline

According to the database, there were 357 in-hospital deaths patients (15.7%). The NLR was found to have a non-linear relationship with in-hospital death risk according to the RCS model. The NLR value for an OR value across 1 is around 7.32(Fig. 4).

All patients with CKD and CAD were divided into two categories based on the NLR: Q1 (NLR ≤ 7.32), and Q2 (NLR > 7.32). Additional file 2: Table S4 illustrates the univariable and multivariable logistic regression analyses results. After accounting for all influencing factors (Table 2, Model 3), the findings from multivariable logistic regression demonstrated that the NLR raised the risk of in-hospital mortality (OR 2.122, 95% CI 1.542–2.921, *P* < 0.001). When NLR was used as a continuous variable, it still increased the risk of hospital death (OR 1.027, 95% CI 1.016–1.039, *P* < 0.001).

**Table 2:** The association between various NLR groups and in-hospital mortality

	Model1	Model2	Model3
NLR	1.047 (1.036–1.057)	1.046 (1.036–1.056)	1.027 (1.016–1.039)
NLR			
< 7.32	Ref	Ref	Ref
≥ 7.32	3.639 (2.816–4.702)	3.598 (2.782–4.653)	2.122 (1.542–2.921)
*P*-Value	< 0.001	< 0.001	< 0.001
Model2	Adjusted for Age, Gender		
Model3	Adjusted for age, gender, sbp_min, spo2_min, ast_max, phosphate_max, wbc_mean, SOFA, glucose_min, bun_min, platelet_mean, pt_min, ptt_mean, ACS		

*NLR* neutrophil-to-lymphocyte ratio, *ACS* acute coronary syndrome, *max* maximum, *min* minimum, *ast* aspartate aminotransferase, *pt* prothrombin time, *SOFA* sequential organ failure assessment, *wbc* white blood cell, *sbp* systolic blood pressure, *ptt* partial thromboplastin time, *bun* blood urea nitrogen

### Subgroup analysis

To validate the correlation between the NLR and in-hospital death, subgroup analyses were performed based on age, gender, SOFA score, and medical conditions such as ACS, diabetes, and dialysis. The interaction between the NLR and in-hospital deaths was influenced by age and SOFA score, whereas gender, diabetes, ACS, and dialysis did not exhibit any interaction with this relationship (Table 3). However, the association between NLR and in-hospital death needed to be further explored (*P* > 0.05).

**Table 3:** The subgroup analysis result of multivariable-adjusted OR for association between NLR and hospital mortality

	Case	TOTAL	Q1	Q2	*P* for trend	*P* for interaction
Age						
≥ 75	215	1133	Ref	2.645 (1.732–4.040)	0.001	0.001
< 75	142	1121	Ref	1.404 (0.826–2.384)	0.21	
Gender						
Male	236	1529	Ref	2.205 (1.481–3.283)	0.001	0.503
Female	121	725	Ref	1.971 (1.135–3.424)	0.016	
Diabetes						
Yes	199	1320	Ref	1.739 (1.141–2.651)	0.01	0.428
No	158	934	Ref	3.023 (1.806–5.059)	0.001	
ACS						
Yes	110	644	Ref	1.409 (0.807–2.460)	0.228	0.179
No	247	1610	Ref	2.523 (1.693–3.760)	0.001	
Dialysis						
Yes	122	455	Ref	1.848 (0.980–3.483)	0.058	0.57
No	235	1799	Ref	2.109 (1.434–3.100)	0.001	
SOFA						
≤ 6	89	1249	Ref	2.383 (1.412–4.020)	0.001	0.017
> 6	268	1005	Ref	1.862 (1.233–2.811)	0.003	

*NLR* neutrophil-to-lymphocyte ratio, *ACS* acute coronary syndrome

### NLR and traditional cardiovascular scores

**Fig. 5:**
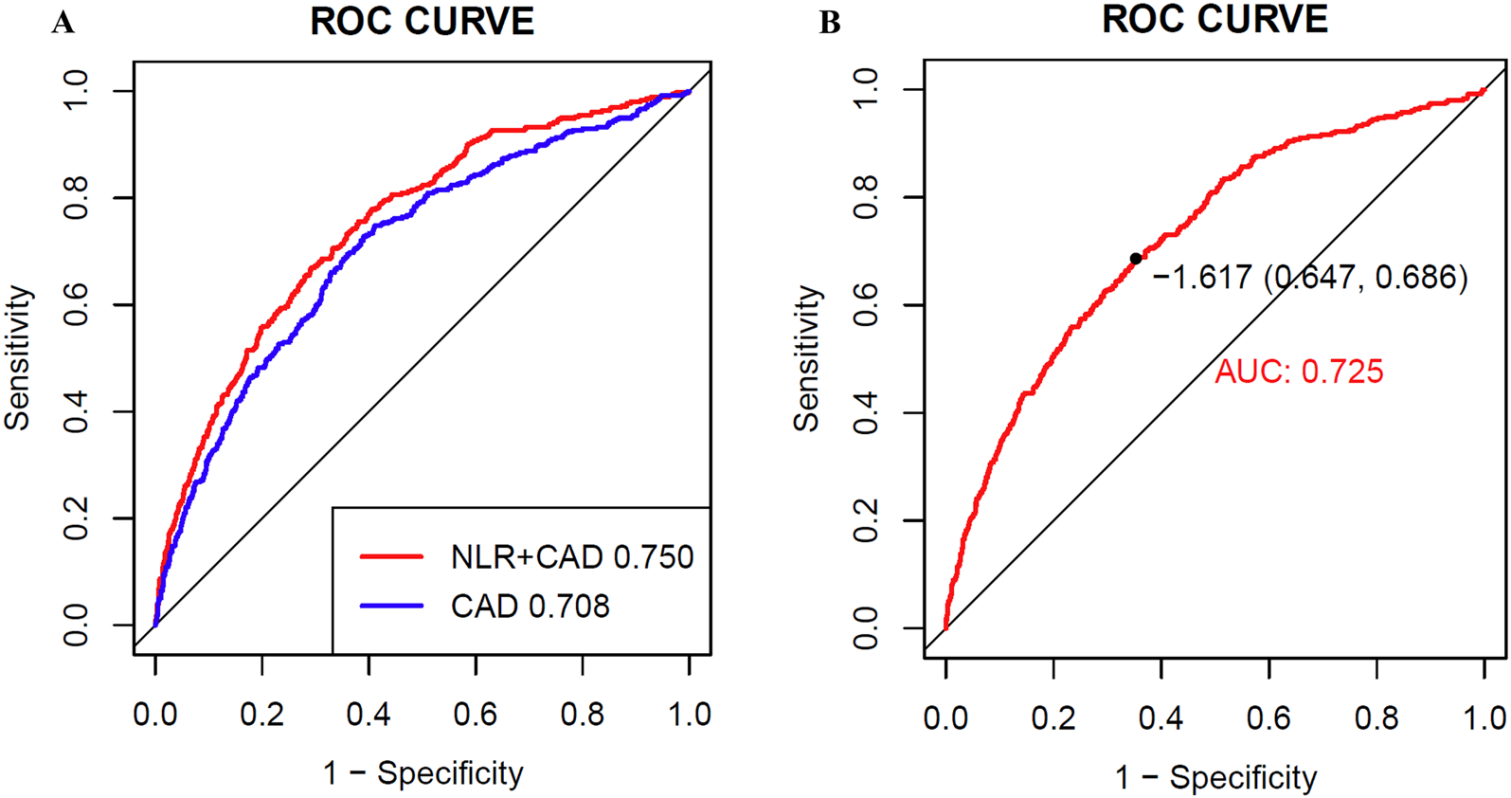
The predictive value of NLR. The combined model (**A**) which added NLR to traditional risk factors had a more accurate prediction (AUC 0.750) than traditional risk factors model (AUC 0.708, *P* < 0.001 by DeLong’s test). NLR had good predictive values in the external database (**B**). AUC, area under curves; NLR, neutrophil-to-lymphocyte ratio

The ROC analysis exhibited that NLR combined with traditional cardiovascular risk factors improved the predictive value for hospital mortality in the CKD and CAD patients in ICU (Fig. 5A). To be specific, the combined model which added NLR to traditional risk factors had a more accurate prediction (AUC 0.750) than traditional risk factors model (AUC 0.708, *P* < 0.001 by DeLong’s test).

### External validation

The study included 1119 patients from eICU-CRD, based on the same criteria as training set. The median NLR was 7.29 (4.4, 11.8). 188 individuals (16.8%) passed away during their hospital stay. The baseline characteristics are summarized in Additional file 2: Table S5. NLR had good predictive values in the external database (AUC = 0.725, Fig. 5B).

## Discussion

The study provided strong evidence that supports the significance of NLR as a predictive factor for in-hospital death among patients who simultaneously had CAD and CKD in the ICU environment. The findings underscored that, within a specific range, an elevated NLR was indicative of an increased likelihood of in-hospital mortality among individuals with both CKD and CAD. Significantly, even after careful control for possible confounding factors, the connection between NLR and increased mortality during hospitalization remained statistically significant.

The role of inflammation is crucial in starting, advancing, and destabilizing atherosclerotic plaques. There is a strong consensus that systemic inflammation is associated with inflammation in the vascular walls [21]. The activation of lymphocytes and monocytes is crucial in the early phases of atherosclerosis, whereas neutrophils are implicated in plaque destabilization and thrombosis [22]. Importantly, the NLR functions as an easily accessible biomarker that indicates inflammation in the vascular wall [23], and its predictive importance has been recorded in different cardiovascular disorders [24]. Elevated NLR levels have been linked to an augmented risk of atrial arrhythmia [25–27], ventricular arrhythmia [28], and adverse outcomes in acute decompensated heart failure [29], acute coronary syndromes [30] and CKD [31], and overall mortality [32] in diverse populations.

CKD represents a persistent inflammatory state, with inflammation being recognized as a crucial catalyst for progressive tubule-interstitial fibrosis, ultimately leading to end-stage renal disease (ESRD) [33]. Numerous investigations have underscored the pivotal role of inflammation in the trajectory of declining kidney function [34]. Notably, anti-inflammatory interventions targeting tubulointerstitial fibrosis in CKD have shown promise in conferring renal protective effects [35]. Extensive research has further elucidated the prognostic value of inflammatory markers in the context of CKD, with studies revealing that an elevation in neutrophil count coupled with a reduction in lymphocyte counts serves as a predictive indicator for mortality in both hemodialysis and peritoneal dialysis patients [36]. Additionally, the NLR has emerged as a valuable metric, reflecting the progression rate from stage 4 chronic kidney disease to the necessity for dialysis [37].

However, there is a paucity of data substantiating the associations between NLR and the mortality risk in individuals afflicted with both CKD and CAD. Based on our current understanding, this research is the first investigation that examines the connection between NLR and in-hospital death in ICU patients who have both CAD and CKD, using a considerable number of participants.

After conducting a multivariate regression analysis and then performing a subgroup analysis, our results confirmed that there is a significant and independent connection between NLR and the likelihood of in-hospital death in patients who have been diagnosed with both CKD and CAD [38]. Alternatively, it was conceivable that the immune system underwent compromise as an integral component of CKD, and NLR served as a surrogate marker for this intricate biological phenomenon.

In our subgroup analysis, it was observed that the non-linear association between NLR and in-hospital mortality among patients with both CAD and CKD in the ICU remained consistent across various subgroups, including elderly individuals, both male and female cohorts, those with and without diabetes, those without acute coronary syndrome (non-ACS), non-dialysis patients, and those with different ranges of Sequential Organ Failure Assessment (SOFA) scores. Furthermore, a significant interaction effect was identified with age and SOFA score (*P* for interaction < 0.05). Our findings indicated that a heightened NLR in elderly patients with CAD and CKD in the ICU was associated with an increased risk of in-hospital mortality. This observed association may be elucidated by the potential mechanism wherein the aging process contributes to immune system compromise, and the NLR serves as a surrogate marker for this intricate biological phenomenon [32]. The SOFA score, initially termed Sepsis-related Organ Failure Assessment, was introduced by The Working Group on Sepsis-Related Problems in 1996 [39]. This scoring system evaluates the functioning of six organ systems, assigning scores ranging from 0 (indicating no organ dysfunction) to 4 (indicating severe organ dysfunction). The cumulative score, derived by summing individual organ scores, ranges from 0 to 24. Numerous studies have consistently demonstrated its efficacy in predicting mortality among cohorts of critically ill patients [40–43]. We observed a significant interaction between the SOFA score and the NLR in patients with both CKD and CAD. This interaction was found to be associated with the prognosis of the patients.

Previous studies have indicated that NLR plays a very crucial role in patients with renal insufficiency, especially in the ICU. In patients with acute kidney injury admitted to the ICU, NLR was shown to be an independent predictor of accelerated progression of acute kidney injury and in-hospital mortality. A large cohort study demonstrated a J-shaped association between acute kidney injury progression and NLR [44]. Furthermore, in patients with end-stage renal disease, i.e., hemodialysis status, NLR was significantly associated with higher erythropoietic resistance, whereas platelet lymphocyte ratio was not associated with erythropoietin responsiveness reflected by erythropoietic resistance, which demonstrated the important predictive role of NLR in the clinical setting [45]. Further, a small sample size CKD cohort study showed that higher NLR reflected the advanced stage of CKD, suggesting that NLR could play a role as a biomarker for predicting CKD progression [46]. However, the role of NLR in the CKD combined with CAD population with higher mortality has not been explored.

Our findings bore significant implications for clinical practice and patient care. Within our investigation, an elevated NLR emerged as a discernible factor linked to heightened in-hospital mortality among individuals concurrently diagnosed with CAD and CKD within a defined spectrum. This observation underscored the potential utility of NLR as a pertinent instrument for risk stratification and management within this inherently high-risk patient cohort. To address the augmented risk associated with elevated NLR levels, a comprehensive risk management strategy was warranted. This strategy should have encompassed assertive interventions targeting cardiovascular risk factors, including meticulous control of lipid levels, blood pressure regulation, and support for smoking cessation. Regular monitoring coupled with timely interventions in cases of elevated NLR levels assumed pivotal significance in mitigating the incidence of adverse outcomes.

The present investigation marked the inaugural exploration into the role of the NLR in patients simultaneously afflicted with CKD and CAD within the ICU setting. This study underscored the imperative for a comprehensive risk management strategy in individuals grappling with the dual burden of CAD and CKD, with the NLR emerging as a pivotal instrument in this paradigm. Nevertheless, it is essential to acknowledge the limitations inherent in our findings, which are influenced by several factors. First, the study is primarily constrained by a modest sample size. It is imperative for future research endeavors to prioritize larger sample sizes, thereby enhancing the robustness of evidence needed to substantiate our findings. Second, the retrospective nature of this study is notable, prompting considerations regarding the potential impact of confounding variables and selection bias on the observed outcomes. Moreover, the highest absolute NEU and LYM could originate from different blood tests. The NLR value in this study were calculated based on the highest absolute NEU and LYM values recorded throughout the patient’s stay in the hospital. The NEU count will necessarily increase and LYM count will decrease in a patient with worsening clinic generally. Therefore, it impractical to obtain both the highest NEU and LYM values in the same tube of blood sample. The relationship between the NLR ratio at admission and prognosis needs to be further explored, limited by the natural shortcomings of public databases. Further, it is essential to acknowledge that the dataset employed in this study may lack crucial clinical details, including the patient’s dialysis status, which could potentially influence inflammatory status. To address these constraints, upcoming research endeavors should strive to encompass a comprehensive spectrum of clinical information and systematically monitor the longitudinal fluctuations in NLR values.

## Conclusions

As a result, our research showcased the prognostic significance of NLR in predicting the likelihood of in-hospital death among ICU patients suffering from both CAD and CKD. In this high-risk cohort, NLR emerged as a potentially valuable instrument for risk stratification and subsequent management. Further research is necessary to confirm these results and clarify the underlying mechanisms that govern the link between NLR and mortality in individuals with both CAD and CKD.

### Supplementary Information

**Additional file 1: Materials S1.** R code for feature selection.

**Additional file 2: Table S1.** Baseline characteristics for patients included in the study divided by in-hospital survival status. (Full version). **Table S2.** Feature selection for the relationship between various NLR indices and in-hospital mortality analyzed by the Boruta algorithm. **Table S3.** Top 30 important variables for the relationship between NLR and in-hospital mortality according to three methods. **Table S4.** The univariable and multivariable for all identified covariates as risk factor for in-hospital mortality. **Table S5.** Baseline characteristics for external validation.

**Additional file 3: Figure S1.** Feature selection for the relationship between NLR and in-hospital mortality based on root mean square error (RMSE) loss after permutations.

## Data Availability

The data supporting this study’s findings are available from the Medical Information Mart for Intensive Care IV (MIMIC IV), but restrictions apply to the availability of these data, which were used under license for the current study, and so are not publicly available. Data are, however, available from the author Jingjing Luo (luojingjing677@foxmail.com) upon reasonable request and with permission of MIMIC.

## Abbreviations

NLR: Neutrophil-to-lymphocyte ratio
CKD: Chronic kidney disease
CAD: Coronary artery disease
MIMIC-IV: Medical Information Mart for Intensive Care IV
LR: Logistic regression
ESRD: End stage renal disease
ML: Machine learning
ICD-9: International Classification of Diseases and Ninth Revision
ICD-10: International Classification of Diseases and tenth Revision
ICU: Intensive care unit
scr: Serum creatinine
eGFR: Estimated glomerular filtration rate
ACS: Acute coronary syndrome
HT: Hypertension
PCI: Percutaneous coronary intervention
CABG: Coronary artery bypass grafting
NOAC: Non-vitamin K antagonist oral anticoagulant
CRRT: Continuous renal replacement therapy
max: Maximum
min: Minimum
WBC: White blood cell
RBC: Red blood cell
ALT: Alanine aminotransferase
AST: Aspartate aminotransferase
ALP: Alkaline phosphatase
BUN: Blood urea nitrogen
INR: International normalized ratio
PT: Prothrombin time
PTT: Partial thromboplastin time
SOFA: Sequential organ failure assessment
sbp: Systolic blood pressure
dbp: Diastolic blood pressure
mbp: Mean blood pressure
HR: Heart rate
SpO_2_: Oxyhemoglobin saturation
RMSE: Root mean square error
%IncMSE: The percentage of the increase in mean square error
IncNodePurity: The increase in node purity
NEU: Neutrophils
LYM: Lymphocytes
ROC: Receiver operating characteristic
AUC: Area under curves
BMI: Body mass index
eICU-CRD: The Telehealth Intensive Care Unit Collaborative Research Database
