# Supplementary material for: Association between the neutrophil-to-lymphocyte ratio and in-hospital mortality in patients with chronic kidney disease and coronary artery disease in the intensive care unit
Source: Eur J Med Res. 2024 Apr 30;29:260. doi: 10.1186/s40001-024-01850-3 (PMC11059689; doi:10.1186/s40001-024-01850-3)
Supplement: Supplementary file 2 — Additional file 2: Table S1. Baseline characteristics for patients included in the study divided by in-hospital survival status. (Full version). Table S2. Feature selection for the relationship between various NLR indices and in-hospital mortality analyzed by the Boruta algorithm. Table S3. Top 30 important variables for the relationship between NLR and in-hospital mortality according to three methods. Table S4. The univariable and multivariable for all identified covariates as risk factor for in-hospital mortality. Table S5. Baseline characteristics for external validation. [file 40001_2024_1850_MOESM2_ESM.pdf]

Table S1 baseline characteristic for patients included in the study divided by in-hospital situation (full version)

	Overall	Survivor	Non-survivor	*P*-Value
n	2254	1897	357	
age	76.0 [68.0,83.0]	75.0 [67.0,82.0]	79.0 [70.0,85.0]	<0.001
Female, n (%)	725 (32.2)	604 (31.8)	121 (33.9)	0.484
los_icu	2.6 [1.3,4.8]	2.5 [1.3,4.3]	3.3 [1.3,6.7]	0.004
scr_baseline	1.4 [1.1,2.0]	1.3 [1.0,1.9]	1.6 [1.1,2.5]	<0.001
myocardial_infarct, n (%)	1506 (66.8)	1229 (64.8)	277 (77.6)	<0.001
congestive_heart_failure, n (%)	1476 (65.5)	1212 (63.9)	264 (73.9)	<0.001
peripheral_vascular_disease, n (%)	515 (22.8)	422 (22.2)	93 (26.1)	0.133
cerebrovascular_disease, n (%)	352 (15.6)	289 (15.2)	63 (17.6)	0.284
dementia, n (%)	115 (5.1)	94 (5.0)	21 (5.9)	0.549
chronic_pulmonary_disease, n (%)	674 (29.9)	561 (29.6)	113 (31.7)	0.469
rheumatic_disease, n (%)	97 (4.3)	84 (4.4)	13 (3.6)	0.596
peptic_ulcer_disease, n (%)	71 (3.1)	56 (3.0)	15 (4.2)	0.282
diabetes, n (%)	907 (40.2)	784 (41.3)	123 (34.5)	0.018
malignant_cancer, n (%)	223 (9.9)	167 (8.8)	56 (15.7)	<0.001
mild_liver_disease, n (%)	188 (8.3)	133 (7.0)	55 (15.4)	<0.001
severe_liver_disease, n (%)	55 (2.4)	34 (1.8)	21 (5.9)	<0.001
atrial_fibrillation_or_flutter, n (%)	1083 (48.0)	876 (46.2)	207 (58.0)	<0.001
ventricular_arrhythmia, n (%)	139 (6.2)	86 (4.5)	53 (14.8)	<0.001
cardiac_arrest, n (%)	110 (4.9)	60 (3.2)	50 (14.0)	<0.001
ACS, n (%)	644 (28.6)	534 (28.1)	110 (30.8)	0.338
PCI, n (%)	88 (3.9)	75 (4.0)	13 (3.6)	0.896
CABG, n (%)	465 (20.6)	451 (23.8)	14 (3.9)	<0.001
aspirin, n (%)	1926 (85.4)	1651 (87.0)	275 (77.0)	<0.001
clopidogrel, n (%)	641 (28.4)	550 (29.0)	91 (25.5)	0.2
ticagrelor, n (%)	2 (0.1)	1 (0.1)	1 (0.3)	0.292
statin, n (%)	1888 (83.8)	1646 (86.8)	242 (67.8)	<0.001
beta_blocker, n (%)	1690 (75.0)	1484 (78.2)	206 (57.7)	<0.001
noac, n (%)	171 (7.6)	156 (8.2)	15 (4.2)	0.012
warfarin, n (%)	610 (27.1)	557 (29.4)	53 (14.8)	<0.001
inhospital_hemodialysis, n (%)	166 (7.4)	122 (6.4)	44 (12.3)	<0.001
RRT_inhospital, n (%)	402 (17.8)	298 (15.7)	104 (29.1)	<0.001
troponin_max	0.3 [0.1,1.1]	0.2 [0.1,0.9]	0.6 [0.2,2.1]	<0.001
troponin_min	0.1 [0.0,0.5]	0.1 [0.0,0.4]	0.2 [0.1,0.9]	<0.001
troponin_mean	0.2 [0.1,0.8]	0.2 [0.1,0.7]	0.5 [0.1,1.6]	<0.001
wbc_max	15.6 [11.6,20.9]	15.2 [11.4,20.0]	19.8 [14.3,26.5]	<0.001
wbc_min	6.9 [5.2,8.8]	6.7 [5.2,8.4]	8.4 [6.0,12.0]	<0.001
wbc_mean	10.6 [8.3,13.3]	10.2 [8.1,12.8]	13.1 [10.0,17.6]	<0.001
rbc_max	3.7 [3.3,4.2]	3.7 [3.4,4.2]	3.7 [3.2,4.2]	0.016
rbc_min	2.7 [2.4,3.1]	2.7 [2.4,3.1]	2.6 [2.3,3.2]	0.073
rbc_mean	3.2 [2.9,3.6]	3.2 [2.9,3.6]	3.1 [2.8,3.6]	0.102

hemoglobin_max	11.1 [9.9,12.4]	11.1 [10.0,12.5]	10.8 [9.7,12.1]	0.004
hemoglobin_min	7.9 [7.0,9.3]	8.0 [7.0,9.3]	7.8 [6.7,9.3]	0.012
hemoglobin_mean	9.4 [8.6,10.5]	9.5 [8.6,10.5]	9.3 [8.4,10.4]	0.02
hematocrit_max	34.3 [31.0,38.2]	34.4 [31.0,38.2]	33.9 [30.6,37.8]	0.119
hematocrit_min	24.5 [21.8,28.6]	24.4 [21.9,28.6]	24.5 [21.2,29.0]	0.407
hematocrit_mean	28.9 [26.6,32.1]	29.0 [26.7,32.1]	28.8 [26.4,32.6]	0.548
platelet_max	260.0 [194.0,345.0]	263.0 [199.0,350.0]	236.0 [158.0,317.0]	<0.001
platelet_min	129.0 [91.0,178.0]	131.0 [96.0,180.0]	115.0 [60.0,166.0]	<0.001
platelet_mean	186.0 [142.6,243.6]	189.4 [146.8,246.3]	165.5 [110.3,231.4]	<0.001
alt_max	29.0 [17.0,69.0]	26.0 [16.0,52.0]	76.0 [29.0,316.0]	<0.001
alt_min	18.0 [12.0,30.0]	17.0 [12.0,28.0]	23.0 [13.0,50.0]	<0.001
alt_mean	24.5 [15.0,49.4]	22.0 [14.6,42.0]	46.5 [22.3,160.0]	<0.001
ast_max	44.5 [25.0,109.0]	39.0 [24.0,85.0]	136.0 [47.0,668.0]	<0.001
ast_min	25.0 [18.0,37.0]	24.0 [17.0,34.0]	33.0 [20.0,72.0]	<0.001
ast_mean	35.0 [22.2,67.7]	32.0 [21.2,54.4]	78.5 [36.2,318.0]	<0.001
alp_max	96.0 [71.0,141.8]	93.0 [69.0,132.0]	131.0 [87.0,210.0]	<0.001
alp_min	76.0 [57.0,101.0]	74.0 [56.0,98.0]	82.0 [62.0,122.0]	<0.001
alp_mean	87.1 [67.0,117.8]	84.3 [65.0,112.6]	102.0 [78.1,164.4]	<0.001
bilirubin_total_max	0.6 [0.4,1.1]	0.6 [0.4,1.0]	1.1 [0.6,2.6]	<0.001
bilirubin_total_min	0.4 [0.3,0.7]	0.4 [0.3,0.6]	0.6 [0.3,1.0]	<0.001
bilirubin_total_mean	0.5 [0.4,0.8]	0.5 [0.3,0.8]	0.8 [0.5,1.6]	<0.001
creatinine_max	2.5 [1.7,4.3]	2.3 [1.7,3.9]	3.6 [2.5,5.2]	<0.001
creatinine_min	1.4 [1.1,2.0]	1.3 [1.0,1.9]	1.6 [1.1,2.5]	<0.001
creatinine_mean	1.9 [1.4,2.9]	1.8 [1.4,2.7]	2.4 [1.8,3.7]	<0.001
bun_max	56.0 [38.0,82.0]	52.0 [36.0,77.0]	76.0 [52.0,106.0]	<0.001
bun_min	24.0 [17.0,35.0]	23.0 [16.0,32.0]	31.0 [20.0,50.0]	<0.001
bun_mean	39.2 [27.6,55.2]	37.1 [26.9,51.9]	52.5 [36.9,73.1]	<0.001
potassium_max	5.1 [4.7,5.7]	5.1 [4.7,5.6]	5.4 [4.9,6.0]	<0.001
potassium_min	3.6 [3.3,3.9]	3.6 [3.3,3.9]	3.5 [3.2,3.9]	0.054
potassium_mean	4.3 [4.0,4.5]	4.3 [4.0,4.5]	4.3 [4.1,4.7]	0.001
sodium_max	143.0 [140.0,145.0]	143.0 [140.0,145.0]	143.0 [139.0,147.0]	0.175
sodium_min	134.0 [131.0,137.0]	134.0 [131.0,137.0]	133.0 [129.0,137.0]	0.003
sodium_mean	138.2 [135.9,140.5]	138.3 [136.0,140.4]	138.1 [134.5,141.2]	0.22
total_calcium_max	9.1 [8.7,9.6]	9.1 [8.7,9.6]	9.1 [8.6,9.8]	0.844
total_calcium_min	7.9 [7.5,8.3]	8.0 [7.6,8.4]	7.7 [7.1,8.2]	<0.001
total_calcium_mean	8.5 [8.2,8.9]	8.6 [8.2,8.9]	8.4 [8.0,8.8]	<0.001
free_calcium_max	1.2 [1.1,1.3]	1.2 [1.1,1.3]	1.2 [1.1,1.2]	0.001
free_calcium_min	1.1 [1.0,1.1]	1.1 [1.0,1.1]	1.0 [0.9,1.1]	<0.001
free_calcium_mean	1.1 [1.1,1.2]	1.1 [1.1,1.2]	1.1 [1.1,1.1]	<0.001
magnesium_max	2.6 [2.3,3.0]	2.6 [2.3,3.0]	2.6 [2.4,2.9]	0.656
magnesium_min	1.8 [1.6,2.0]	1.8 [1.6,2.0]	1.8 [1.6,2.0]	0.884
magnesium_mean	2.2 [2.0,2.3]	2.2 [2.0,2.3]	2.2 [2.0,2.4]	0.253
phosphate_max	5.1 [4.2,6.6]	4.9 [4.1,6.1]	6.8 [5.4,8.3]	<0.001
phosphate_min	2.7 [2.2,3.3]	2.7 [2.2,3.3]	2.9 [2.0,4.2]	0.009

phosphate_mean	3.8 [3.3,4.5]	3.8 [3.3,4.4]	4.6 [3.6,5.6]	<0.001
inr_max	1.5 [1.3,2.4]	1.5 [1.3,2.2]	2.0 [1.5,3.4]	<0.001
inr_min	1.1 [1.0,1.2]	1.1 [1.0,1.2]	1.2 [1.1,1.5]	<0.001
inr_mean	1.3 [1.2,1.6]	1.3 [1.1,1.5]	1.6 [1.3,2.1]	<0.001
pt_max	16.8 [14.2,25.5]	16.2 [13.9,23.9]	22.1 [16.3,36.1]	<0.001
pt_min	12.4 [11.4,13.7]	12.2 [11.3,13.3]	13.6 [12.1,16.1]	<0.001
pt_mean	14.2 [12.7,17.7]	14.0 [12.6,16.7]	17.2 [14.1,22.5]	<0.001
ptt_max	53.0 [33.1,114.2]	47.6 [32.4,104.5]	79.7 [41.3,148.5]	<0.001
ptt_min	27.2 [25.1,30.2]	27.0 [25.0,29.7]	29.1 [25.9,34.7]	<0.001
ptt_mean	37.3 [29.8,55.0]	35.6 [29.4,52.9]	47.6 [34.3,63.0]	<0.001
glucose_max	203.0 [157.0,286.0]	196.0 [154.0,275.0]	242.0 [179.0,326.0]	<0.001
glucose_min	86.0 [72.0,101.0]	85.0 [72.0,99.0]	88.0 [71.0,117.0]	0.003
glucose_mean	132.8 [113.2,164.0]	129.9 [112.2,159.4]	153.8 [124.0,195.8]	<0.001
SOFA	6.0 [4.0,9.0]	6.0 [4.0,8.0]	10.0 [7.0,13.0]	<0.001
BMI	28.1 [24.4,32.8]	28.1 [24.5,32.8]	28.0 [24.0,33.3]	0.703
sbp_max	155.0 [140.0,172.0]	155.0 [141.0,172.0]	152.0 [134.0,168.0]	<0.001
sbp_min	84.0 [73.0,93.0]	85.0 [76.0,95.0]	70.0 [54.0,83.0]	<0.001
sbp_mean	116.8 [107.7,127.6]	118.3 [109.6,128.7]	107.4 [99.9,118.1]	<0.001
dbp_max	93.5 [80.0,110.0]	93.0 [80.0,109.0]	94.0 [78.0,111.0]	0.704
dbp_min	39.0 [32.0,45.0]	40.0 [33.0,46.0]	33.0 [26.0,41.0]	<0.001
dbp_mean	58.2 [52.5,64.3]	58.6 [53.0,64.7]	56.3 [50.2,62.2]	<0.001
mbp_max	108.0 [96.0,126.0]	108.0 [96.0,125.0]	109.0 [94.0,133.0]	0.376
mbp_min	52.0 [45.0,59.0]	54.0 [47.0,60.0]	44.0 [31.0,54.0]	<0.001
bmp_mean	74.5 [69.3,80.4]	75.1 [70.1,80.8]	70.8 [66.2,76.6]	<0.001
hr_mx	104.0 [90.0,122.0]	102.0 [89.0,119.0]	116.0 [99.0,137.0]	<0.001
hr_min	62.0 [55.0,70.0]	62.0 [55.0,70.0]	60.0 [50.0,71.0]	0.01
hr_mean	80.2 [71.9,89.2]	79.3 [71.6,87.9]	87.3 [75.5,97.0]	<0.001
spo2_max	1962 (87.0)	1647 (86.8)	315 (88.2)	0.526
spo2_min	90.0 [85.0,92.0]	90.0 [87.0,93.0]	83.0 [74.0,90.0]	<0.001
spo2_mean	96.8 [95.6,97.8]	96.8 [95.7,97.8]	96.7 [94.9,98.0]	0.088
NLR	7.3 [4.4,12.1]	6.7 [4.1,10.9]	11.5 [7.4,19.1]	<0.001
LY_abs_max	1.4 [0.9,2.0]	1.4 [0.9,2.0]	1.1 [0.8,1.8]	<0.001
LY_abs_min	1.0 [0.6,1.6]	1.1 [0.7,1.6]	0.7 [0.4,1.1]	<0.001
LY_abs_mean	1.2 [0.8,1.7]	1.2 [0.8,1.8]	0.9 [0.6,1.4]	<0.001
MONO_abs_max	0.7 [0.4,1.1]	0.7 [0.4,1.0]	1.0 [0.6,1.5]	<0.001
MONO_abs_min	0.5 [0.3,0.8]	0.5 [0.3,0.8]	0.6 [0.3,1.0]	0.001
MONO_abs_mean	0.6 [0.4,0.9]	0.6 [0.4,0.9]	0.8 [0.5,1.2]	<0.001
NEU_abs_max	10.0 [6.8,14.3]	9.5 [6.5,13.5]	13.8 [9.7,20.0]	<0.001
NEU_abs_min	7.7 [5.2,10.9]	7.4 [5.1,10.4]	9.7 [6.3,13.9]	<0.001
NEU_abs_mean	9.0 [6.3,12.5]	8.6 [6.0,11.9]	11.8 [8.1,16.6]	<0.001
LYM_max	12.9 [8.2,19.8]	13.8 [9.0,20.3]	8.9 [5.9,13.4]	<0.001
LYM_min	9.1 [5.1,15.0]	10.2 [6.0,16.0]	5.2 [3.0,8.5]	<0.001
LYM_mean	11.1 [7.1,17.0]	12.0 [7.8,18.1]	7.1 [4.5,10.6]	<0.001
MONO_max	6.4 [4.2,9.4]	6.3 [4.2,9.2]	7.0 [4.8,10.0]	0.003

MONO_min	4.5 [2.9,7.0]	4.5 [2.9,7.0]	4.3 [2.1,6.6]	0.047
MONO_mean	5.5 [3.8,7.9]	5.5 [3.8,7.9]	5.7 [3.9,8.2]	0.344
NEU_max	82.0 [74.6,88.0]	81.0 [73.8,87.1]	86.3 [80.4,90.8]	<0.001
NEU_min	76.8 [68.7,83.2]	76.0 [68.0,82.4]	80.1 [72.0,86.4]	<0.001
NEU_mean	79.3 [72.5,84.9]	78.5 [71.7,84.0]	82.9 [77.0,87.9]	<0.001

NLR, neutrophil-to-lymphocyte ratio; scr, serum creatinine; ACS, acute coronary syndrome, max, maximum; min, minimum; alt alanine aminotransferase; ast, aspartate aminotransferase; inr, International Normalized Ratio; pt, prothrombin time; ptt, partial thromboplastin time; SOFA, sequential organ failure assessment; hr, heart rate; spo2, oxyhemoglobin saturation; bun, blood urea nitrogen; NEU, neutrophils; LYM, lymphocytes; abs, absolute.

Table S2 Boruta analysis result for in-hospital mortality

ACS	Rejected
age	Tentative
gender	Rejected
los_icu	Confirmed
scr_baseline	Tentative
eGFRbaseline	Tentative
myocardial_infarct	Rejected
congestive_heart_failure	Rejected
peripheral_vascular_disease	Rejected
cerebrovascular_disease	Rejected
dementia	Rejected
chronic_pulmonary_disease	Rejected
rheumatic_disease	Rejected
peptic_ulcer_disease	Rejected
diabetes_with_cc	Rejected
diabetes_without_cc	Rejected
malignant_cancer	Rejected
mild_liver_disease	Rejected
severe_liver_disease	Rejected
HT	Rejected
atrial_fibrillation_or_flutter	Rejected
ventricular_arrhythmia	Tentative
cardiac_arrest	Confirmed
PCI	Rejected
CABG	Tentative
aspirin	Rejected
clopidogrel	Rejected
ticagrelor	Rejected
statin	Rejected
beta_blocker	Tentative
noac	Rejected
warfarin	Confirmed
inhospital_hemodialysis	Rejected
inhospital_peritoneal_dialysis	Rejected
RRT_inhospital	Rejected
troponin_max	Tentative
troponin_min	Rejected
troponin_mean	Tentative
wbc_max	Confirmed
wbc_min	Confirmed
wbc_mean	Confirmed
rbc_max	Rejected

rbc_min	Rejected
rbc_mean	Rejected
hemoglobin_max	Rejected
hemoglobin_min	Rejected
hemoglobin_mean	Rejected
hematocrit_max	Rejected
hematocrit_min	Rejected
hematocrit_mean	Rejected
platelet_max	Confirmed
platelet_min	Confirmed
platelet_mean	Confirmed
alt_max	Confirmed
alt_min	Confirmed
alt_mean	Confirmed
ast_max	Confirmed
ast_min	Confirmed
ast_mean	Confirmed
alp_max	Confirmed
alp_min	Rejected
alp_mean	Confirmed
bilirubin_total_max	Confirmed
bilirubin_total_min	Confirmed
bilirubin_total_mean	Confirmed
creatinine_max	Confirmed
creatinine_min	Tentative
creatinine_mean	Confirmed
bun_max	Confirmed
bun_min	Confirmed
bun_mean	Confirmed
potassium_max	Rejected
potassium_min	Confirmed
potassium_mean	Confirmed
sodium_max	Confirmed
sodium_min	Confirmed
sodium_mean	Confirmed
total_calcium_max	Tentative
total_calcium_min	Confirmed
total_calcium_mean	Confirmed
free_calcium_max	Rejected
free_calcium_min	Confirmed
free_calcium_mean	Confirmed
magnesium_max	Confirmed
magnesium_min	Rejected
magnesium_mean	Rejected

phosphate_max	Confirmed
phosphate_min	Confirmed
phosphate_mean	Confirmed
inr_max	Confirmed
inr_min	Confirmed
inr_mean	Confirmed
pt_max	Tentative
pt_min	Confirmed
pt_mean	Confirmed
ptt_max	Rejected
ptt_min	Confirmed
ptt_mean	Confirmed
glucose_max	Tentative
glucose_min	Confirmed
glucose_mean	Confirmed
SOFA	Confirmed
BMI	Rejected
sbp_max	Confirmed
sbp_min	Confirmed
sbp_mean	Confirmed
dbp_max	Confirmed
dbp_min	Confirmed
dbp_mean	Confirmed
mbp_max	Confirmed
mbp_min	Confirmed
bmp_mean	Confirmed
hr_mx	Tentative
hr_min	Confirmed
hr_mean	Confirmed
spo2_max	Rejected
spo2_min	Confirmed
spo2_mean	Confirmed
NLR	Confirmed
LY_abs_max	Confirmed
LY_abs_min	Confirmed
LY_abs_mean	Confirmed
MONO_abs_max	Confirmed
MONO_abs_min	Confirmed
MONO_abs_mean	Confirmed
NEU_abs_max	Confirmed
NEU_abs_min	Confirmed
NEU_abs_mean	Confirmed
LYM_max	Confirmed
LYM_min	Confirmed

LYM_mean	Confirmed
MONO_max	Rejected
MONO_min	Rejected
MONO_mean	Rejected
NEU_max	Confirmed
NEU_min	Confirmed
NEU_mean	Confirmed

NLR, neutrophil-to-lymphocyte ratio; scr, serum creatinine; ACS, acute coronary syndrome, max, maximum; min, minimum; alt alanine aminotransferase; ast, aspartate aminotransferase; inr, International Normalized Ratio; pt, prothrombin time; ptt, partial thromboplastin time; SOFA, sequential organ failure assessment; hr, heart rate; spo2, oxyhemoglobin saturation; bun, blood urea nitrogen; NEU, neutrophils; LYM, lymphocytes; abs, absolute.

Table S3 Top 30 important variables according to different methods

%IncMSE	IncNodePurity	RMSE-loss-after-permutations
sbp_min	sbp_min	spo2_min
spo2_min	spo2_min	sbp_min
LYM_min	ast_mean	ast_mean
ast_max	ast_max	LYM_min
ast_mean	wbc_mean	ast_max
NLR	phosphate_max	phosphate_max
phosphate_max	SOFA	wbc_mean
LYM_mean	sbp_mean	LYM_mean
wbc_mean	wbc_min	SOFA
SOFA	ast_min	wbc_min
glucose_min	platelet_mean	sbp_mean
phosphate_mean	phosphate_mean	glucose_min
NEU_max	pt_min	phosphate_mean
bun_min	glucose_min	platelet_mean
platelet_mean	sodium_mean	bun_min
wbc_min	LYM_min	pt_min
sbp_mean	glucose_mean	bun_mean
bun_mean	LYM_mean	glucose_mean
ast_min	spo2_mean	sodium_mean
pt_mean	ptt_mean	ast_min
phosphate_min	ptt_min	NLR
pt_min	phosphate_min	ptt_mean
inr_mean	mbp_min	mbp_min
ptt_min	bun_min	bilirubin_total_mean
alt_max	bilirubin_total_mean	ptt_min
platelet_max	bmp_mean	bilirubin_total_max
platelet_min	hr_min	spo2_mean
ptt_mean	sodium_max	NEU_max
LYM_max	bun_mean	bmp_mean
sodium_mean	bilirubin_total_max	platelet__min

NLR, neutrophil-to-lymphocyte ratio; scr, serum creatinine; ACS, acute coronary syndrome, max, maximum; min, minimum; alt alanine aminotransferase; ast, aspartate aminotransferase; inr, International Normalized Ratio; pt, prothrombin time; ptt, partial thromboplastin time; SOFA, sequential organ failure assessment; hr, heart rate; spo2, oxyhemoglobin saturation; bun, blood urea nitrogen; NEU, neutrophils; LYM, lymphocytes; abs, absolute.

Table S4 The univariable and multivariable Logistic analysis for all identified covariates as risk factor for in-hospital mortality

	univariable			multivariable		
	OR	95%CI	P-vaue	OR	95%CI	P-vaue
ACS	1.137	0.889-1.454	0.307	1.562	1.135-2.151	0.006
age	1.027	1.016-1.038	<0.001	1.039	1.023-1.055	<0.001
gender	0.911	0.717-1.158	0.446	0.828	0.604-1.134	0.239
sbp_min	0.952	0.945-0.958	<0.001	0.977	0.968-0.986	<0.001
spo2_min	0.932	0.921-0.942	<0.001	0.962	0.951-0.973	<0.001
ast_max	1.000	1.000-1.001	<0.001	1.000	1.000-1.000	0.004
phosphate_max	1.409	1.334-1.488	<0.001	1.238	1.154-1.328	<0.001
wbc_mean	1.088	1.060-1.117	<0.001	1.040	1.021-1.060	<0.001
SOFA	1.270	1.230-1.311	<0.001	1.103	1.055-1.154	<0.001
glucose_min	1.011	1.007-1.014	<0.001	1.011	1.006-1.016	<0.001
bun_min	1.028	1.022-1.033	<0.001	1.012	1.004-1.019	0.002
platelet_mean	0.996	0.994-0.997	<0.001	0.997	0.995-0.999	<0.001
pt_min	1.214	1.170-1.259	<0.001	1.138	1.085-1.194	<0.001
ptt_mean	1.027	1.021-1.033	<0.001	1.016	1.008-1.024	<0.001
NLR	1.047	1.036-1.057	<0.001	1.027	1.016-1.039	<0.001

NLR, neutrophil-to-lymphocyte ratio; ACS, acute coronary syndrome; max, maximum; min, minimum; ast, aspartate aminotransferase; pt, prothrombin time; SOFA, sequential organ failure assessment; wbc, white blood cell; sbp, systolic blood pressure; ptt, partial thromboplastin time; bun, blood urea nitrogen.

Table S5 baseline characteristic for external validation

	Overall	Survivor	Non-survivor	*P*-Value
n	1119	931	188	
age	76.00 [67.00, 83.00]	75.00 [66.00, 83.00]	79.00 [70.00, 85.00]	<0.001
Female, n (%)	352 (31.5)	292 (31.4)	60 (31.9)	0.95
los_icu	2.50 [1.33, 4.54]	2.38 [1.33, 4.21]	3.10 [1.21, 6.36]	0.071
scr_baseline	1.40 [1.10, 2.00]	1.40 [1.00, 1.90]	1.50 [1.10, 2.42]	0.005
myocardial_infarct, n (%)	751 (67.1)	600 (64.4)	151 (80.3)	<0.001
ACS, n (%)	319 (28.5)	259 (27.8)	60 (31.9)	0.296
aspirin, n (%)	947 (84.6)	801 (86.0)	146 (77.7)	0.005
clopidogrel, n (%)	310 (27.7)	267 (28.7)	43 (22.9)	0.125
statin, n (%)	933 (83.4)	807 (86.7)	126 (67.0)	<0.001
beta_blocker, n (%)	824 (73.6)	722 (77.6)	102 (54.3)	<0.001
warfarin, n (%)	299 (26.7)	274 (29.4)	25 (13.3)	<0.001
wbc_max	15.80 [11.70, 20.90]	15.20 [11.00, 19.90]	20.20 [14.47, 26.27]	<0.001
rbc_max	3.72 [3.34, 4.18]	3.73 [3.36, 4.18]	3.67 [3.19, 4.19]	0.315
platelet_max	258.00 [191.00, 350.50]	261.00 [197.00, 353.50]	241.50 [165.75, 340.50]	0.005
alt_max	30.00 [17.00, 66.00]	27.00 [16.00, 50.00]	73.50 [27.00, 263.50]	<0.001
ast_max	45.00 [26.00, 103.50]	40.00 [24.00, 81.50]	135.00 [45.50, 563.25]	<0.001
bun_max	56.00 [37.00, 82.00]	52.00 [36.00, 77.00]	76.00 [52.00, 103.50]	<0.001
SOFA	6.00 [4.00, 9.00]	6.00 [4.00, 8.00]	10.00 [6.75, 13.00]	<0.001
BMI	28.34 [24.54, 33.14]	28.23 [24.62, 33.14]	28.47 [24.16, 33.16]	0.929
sbp_max	153.00 [139.00, 171.00]	154.00 [140.00, 171.00]	149.50 [131.75, 169.00]	0.009
dbp_max	92.00 [79.00, 108.00]	92.00 [79.00, 108.00]	92.00 [75.00, 108.50]	0.516
NLR	7.29 [4.27, 11.77]	6.64 [3.98, 10.74]	11.16 [7.28, 17.54]	<0.001
LY_abs_max	1.38 [0.90, 1.99]	1.39 [0.93, 2.00]	1.31 [0.80, 1.93]	0.13
NEU_abs_max	10.07 [6.80, 14.39]	9.49 [6.39, 13.36]	14.14 [10.31, 20.30]	<0.001

NLR, neutrophil-to-lymphocyte ratio; scr, serum creatinine; ACS, acute coronary syndrome, max, maximum; min, minimum; alt alanine aminotransferase; ast, aspartate aminotransferase; inr, International Normalized Ratio; SOFA, sequential organ failure assessment; hr, heart rate; spo2, oxyhemoglobin saturation; bun, blood urea nitrogen; NEU, neutrophils; LYM, lymphocytes; abs, absolute.
