# Supplementary figures and images for: Association between the neutrophil-to-lymphocyte ratio and in-hospital mortality in patients with chronic kidney disease and coronary artery disease in the intensive care unit
Source: Eur J Med Res. 2024 Apr 30;29:260. doi: 10.1186/s40001-024-01850-3 (PMC11059689; doi:10.1186/s40001-024-01850-3)

Feature Importance  
created for the randomForest model  
randomForest

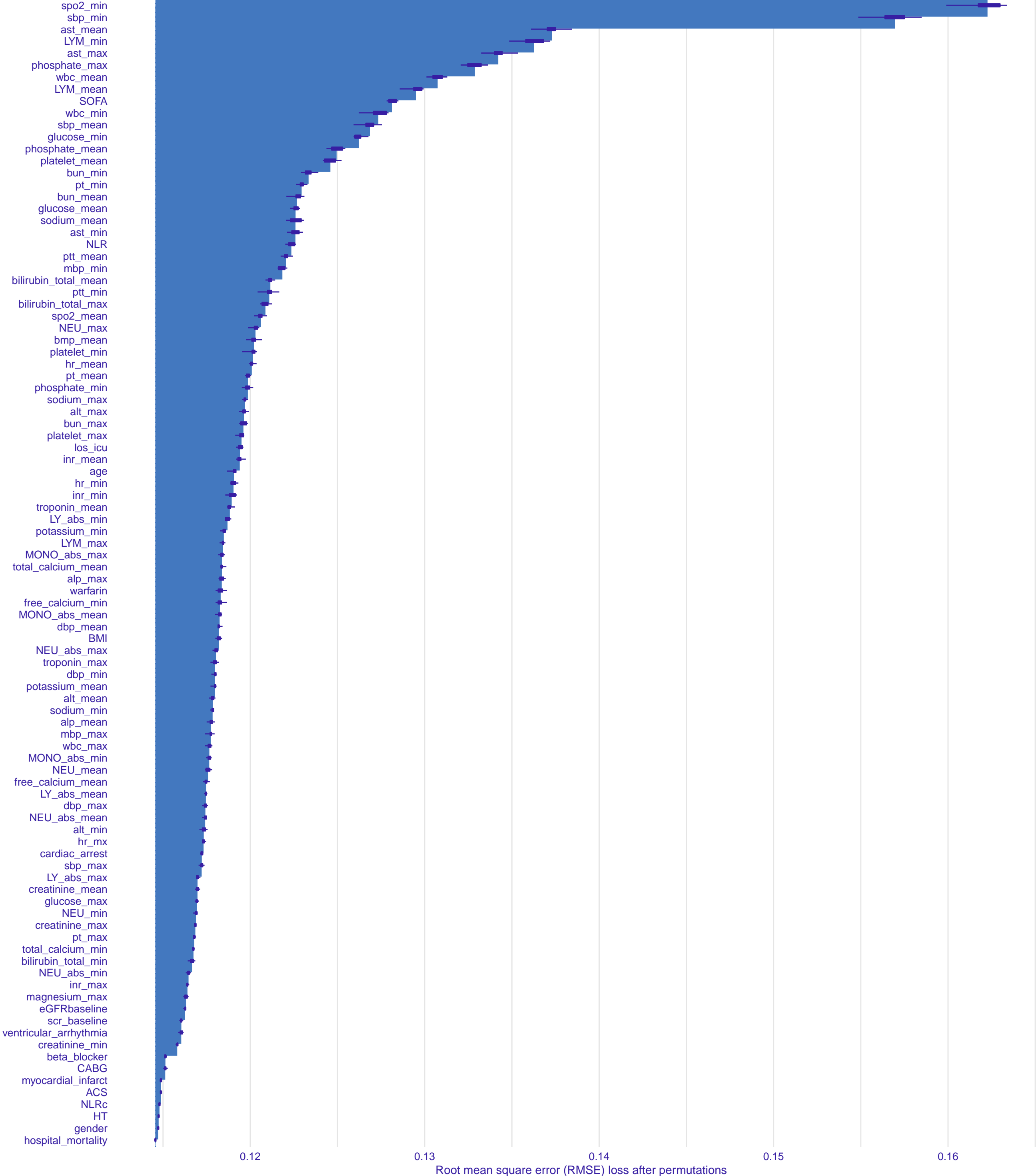

Supplement: Supplementary file 3 — Additional file 3: Figure S1. Feature selection for the relationship between NLR and in-hospital mortality based on root mean square error (RMSE) loss after permutations. [file 40001_2024_1850_MOESM3_ESM.pdf]
